# Effect of Soft Drinks and Fresh Fruit Juice on Surface Roughness of Commonly used Restorative Materials

**DOI:** 10.5005/jp-journals-10005-1274

**Published:** 2015-04-28

**Authors:** Prabhadevi Maganur, V Satish, AR Prabhakar, Srinivas Namineni

**Affiliations:** Associate Professor, Department of Pedodontics, College of Dentistry, Jizan Saudi Arabia; Associate Professor, Department of Pedodontics, College of Dentistry, Jizan Saudi Arabia; Professor and Head, Department of Pedodontics, Bapuji Dental College and Hospital, Davangere, Karnataka, India; Professor and Head, Department of Pedodontics, Sree Sai College of Dental Sciences, Davangere, Karnataka, India

**Keywords:** Soft drinks and fresh fruit juice, surface roughness, Restorative material.

## Abstract

In this *in vitro* study, the effects of a Cola drink, and fresh fruit juice (citrus) on the surface roughness on flowable composite and resin-modified glass ionomer cement (RMGIC) each was evaluated and compared. Using a brass mold 70 pellets each of flowable composite (Filtek™ Flow) and RMGIC tricure restorative material were prepared according to the manufacturer’s instructions. Two groups (groups I and II) were formed containing 30 pellets of each material. Remaining 10 pellets of each restorative material did form the control group [water (group III)]. Experimental group pellets were again divided into three subgroups (mild, moderate and severe) containing 10 pellets each and were kept in plastic containers with 30 ml Cola drink (group I) and fresh fruit juice (group II) respectively. Immersion regime was followed according to M aupome G et al. Baseline and final surface roughness (Ra) value for each pellet was evaluated using a profilometer. Statistical analysis was done with Wilcoxon’s signed rank test and analysis of variance (ANOVA) followed by Mann-Whitney test. Results showed that the erosive effect of both Cola drink and fresh fruit juice caused significant surface roughness on both flowable composite and RMGIC restorative materials in the mild, moderate and severe immersion regimes.

**How to cite this article:** Maganur P, Satish V, Prabhakar AR, Namineni S. Effect of Soft Drinks and Fresh Fruit Juice on Surface Roughness of Commonly used Restorative Materials. Int J Clin Pediatr Dent 2015;8(1):1-5.

## INTRODUCTION

Different forms of destructive processes other than caries affecting the teeth and leading to an irreversible loss of tooth structure from the external surface are described in the literature. They are referred to as abrasion, demastication, attrition, abfraction, resorption and erosion.^1^ Erosion differs from dental caries in that it manifests as an irreversible loss of dental hard tissue by a chemical process that does not involve bacteria. Since, tissue loss is insidious in nature and may not be apparent until the patient reports symptoms of sensitivity or the fracture of thinned incisal edges. Unlike dental caries, erosion occurs on plaque free sites.^1^ Epidemio-logical studies have shown that the prevalence of dental erosion in children varies widely between 2 and 57%.^2^ Soft drinks have a detrimental effect on the teeth upon prolonged exposure.^2,6^ Acidic soft drinks cause enamel demineralization on etched tooth surfaces.^3,4^ High intake of acidic drinks, fruits and liquid medications may constitute possible etiological and/or aggravating factors for severe dental erosion.^5^

Even the primary teeth are highly susceptible to the acidic drinks.^6^ According to studies, deciduous tooth enamel is found to be softer than permanent tooth enamel and hence more susceptible to erosive changes.^5,7^ Factors that cause surface changes on enamel can similarly influence certain properties of the restoration. This clinically jeopardizes the life of the restoration, as the ultimate success of the restorative material is indicated by its longevity in the oral cavity. The effect of the beverages may be stronger, depending on their intrinsic features, such as chemical composition of the restorative materials or external features, such as finishing/polishing of restoration. Moreover, the impact of a beverage on the materials may be directly related to the amount and frequency of its intake.^8^ Restoring such noncarious lesion presents a special clinical challenge, as it requires a restorative material, which can adhere to two different types of tooth tissue. Restorative materials that fulfill the above criteria are more clinically accepted. Resin composites and glass ionomer cements are the materials of choice in restoring such cervical lesions (class V restorations).^9^

## MATERIALS AND METHODS

Cola drink and fresh fruit juice (citrus) were used as experimental drinks and water as control. Restorative materials used for the study were flowable composite and resin-modified glass ionomer cement (RMGIC).

Using a brass mold of inner diameter 4 × 4 mm thickness 70 pellets each of flowable composite (Filtek™ Flow) and RMGIC tricure restorative material were prepared according to the manufacturer’s instructions. Pellets were cured using matrix strips to obtain proper finish of the surface. Two groups (groups I and II) were formed containing 30 pellets of each material. Remaining 10 pellets of each restorative material constituted for control group (water). Experimental group pellets were again divided into three subgroups (mild, moderate, severe) containing 10 pellets each and were kept in plastic containers with 30 ml Cola drink (group I) and fresh fruit juice (group II) respectively. The pellets were rinsed in normal saline before and after each immersion. Apart from the exposure time, pellets were stored in water at room temperature. Immersion regime was followed according to Maupome G et al.^10^ Each immersion period constituted for 5 minutes.

**Table:** 

*Groups*		*Mild immersio (1 time)/ day*		Moderate immersion (5 times)/ day*		Severe immersion (10 times)/ day*	
*Group I: Cola drink*							
I A Flowable composite		10		10		10	
(Filtek™ Flow)							
I B RMGIC		10		10		10	
*Group II: Fresh fruit juice (citrus)*							
II A Flowable composite		10		10		10	
(Filtek™ Flow)							
II B RMGIC		10		10		10	
*Group III: Water (control)*							
III A Flowable composite 10 pellets							
III B RMGIC 10 pellets							

*Both moderate and severe immersion regimes were carried in evenly distributed 12 hours

Baseline surface roughness value and final roughness value of the pellets were recorded through profilometer (Taylor and Hobson, England).

## RESULTS

Flowable composite specimens showed an increased tendency of surface roughness as the number of immersions increased. The results were statistically highly significant (p < 0.001). Analysis of variance (ANOVA) scored a value of F = 487.9. Analysis of variance value of group: I, II and III in mild immersion was F = 75.1, in moderate immersion F = 956.4 and in severe immersion F = 1272.4 respectively; which were statistically highly significant (p < 0.001) (Tables 1 to 3).

Resin-modified glass ionomer cement (RMGIC) samples showed increased surface roughness at different immersion regimes indicating that as the number of immersions increases, the surface roughness also increases. The results were statistically significant (p < 0.001). Analysis of variance scored value of F = 762.1. All the samples scored an increase in the surface roughness as the number of immersions were increased. The results were statistically highly significant (p < 0.001). Analysis of variance scored a value of F = 88.4. The group I specimens compared to groups II and III, in low immersion regime, showed statistically no significant rough ness with p-value of (p = 0.08) and (p = 0.10) respectively. In group II samples compared to group III, in mild immersion regime, the surface roughness was not statistically significant (p = 0.83). In group I samples compared to groups III and II samples compared to group III, in both moderate and severe immersion regimes, the results were statistically highly significant (p < 0.001). In group I samples compared to group II, in moderate, the surface roughness was statistically highly significant (p < 0.001) and in severe immersion regime surface roughness was statistically significant (p < 0.01). Analysis of variance value of groups I, II and III in mild immersion group was F = 5.69 which was statistically significant (p < 0.05). Analysis of variance value of groups I, II and III in moderate and severe immersion regimes were F = 628.7 and F = 217.6 respectively, which was statistically highly significant (p < 0.001) (Tables 4 to 6).

## DISCUSSION

Erosion, derived from the Latin verb ‘Erodere erosi, Erosum’ (to gnaw to corrode) describes the process of gradual destruction of the surface of something, usually by electrolytic or chemical process.^1^ Surveys on dental erosion are few in number with anecdotal case reports. Occurrence of dental erosion has appeared in the literature as case reports as early as late 17th century. Early reports focused on the clinical presentation of erosive lesions in adult population. Later papers concentrated more on the putative etiological factors, such as acidic beverages and fruit juices.^11^ Erosion has a multifactorial etiology, which includes extrinsic and intrinsic factors. The etiological intrinsic factors of dental erosion include upper gastrointestinal disorders, metabolic and endocrine disorder and indirect side effect of drugs, anorexia and bulimia nervosa.^2^ Erosion could occur from food or drinks containing a variety of acidic components present in the food. Most of the soft drinks available in the market today do have phosphoric acid as a common constituent.^12,13^ The acid constituent of the Cola soft drink, which gives a peculiar tangy taste and known for its preservative property, is known to have an established role in the erosive process. Studies have showed that substances in Cola soft drinks absolutely affect the integrity of the enamel surface.^14,15^ These carbonated beverages and sport drinks have a pH below 3.5 and experiments have revealed that enamel dissolution occurs below pH.^4^

**Table 1:** **Table 1:** Descriptive statistics on mean surface roughness values of flowable composite following various immersion regime in different media

		*Mild immersion*						*Moderate immersion*						*Severe immersion*					
*Particulars*		*Before*		*After*		*Difference*		*Before*		*After*		*Difference*		*Before*		*After*		*Difference*	
*Group I: Cola drink*																			
Mean		0.125		0.238		0.114		0.124		0.0562		0.440		0.126		0.847		0.720	
SD		0.001		0.032		0.033		0.004		0.029		0.032		0.002		0.056		0.048	
Ratio		-		-		1.897		-		-		4.56		-		-		6.72	
p-value*		-		-		<0.05 S		-		-		<0.05 S		-		-		<0.05 S	
*Group II: Fresh fruit juice (citrus)*																	
Mean		0.124		0.139		0.015		0.127		0.270		0.143		0.125		0.544		0.419	
SD		0.010		0.015		0.006		0.004		0.016		0.015		0.003		0.005		0.003	
Ratio		-		-		1.13		-		-		2.12		-		-		4.2	
p-value*		-		-		<0.05 S		-		-		<0.05 S		-		-		<0.05 S	
*Group III: Water (control)*																			
*No immersion regime was followed*																			
Mean		0.124		0.126		0.002													
SD		0.003		0.002		0.004													
Ratio		-		-		1.0													
p-value*		-		-		p > 0.05 NS													

*p-value: p < 0.001 highly significant (HS); p < 0.05, p < 0.01 significant (S) and p > 0.05 not significant (NS)

**Table 2:** **Table 2:** Intragroup comparison at different concentrations of various media on flowable composite following various immersion regime

*Groups*		*ANOVA F*		*Mild vs moderate*		*Mild vs severe*		*Moderate vs severe*	
		487.9		p < 0.001		p < 0.001		p < 0.001	
Group I: Cola drink		p < 0.001		HS		HS		HS	
		HS							
		3357.0		p < 0.001		p < 0.001		p < 0.001	
Group II: Fresh Fruit juice (citrus)		p < 0.001		HS		HS		HS	
		HS							

Intragroup comparison: Wilcoxon’s signed rank test; HS: Highly significant

**Table 3:** **Table 3:** Intergroup comparison at different concentrations of various media on flowable composite following various immersion regime

		*ANOVA F*		*75.1, p < 0.001 HS*		*956.4, p < 0.001 HS*		*1272.4, p < 0.001 HS*	
Differences between groups**		Groups I-II		p < 0.001 HS		p < 0.001 HS		p < 0.001 HS	
		Groups I-III		p < 0.001 HS		p < 0.001 HS		p < 0.001 HS	
		Groups II-III		p < 0.01 S		p < 0.001 HS		p < 0.001 HS	

**Intergroup comparison: One-way ANOVA, Mann-Whitney test; S: Significant; HS: Highly significant

The search for an ideal restorative material to replace the tooth tissue with adhesive and caries protective properties together with a simple clinical application procedure have led to the constant development of newer restorative materials. Resin-modified glass ionomer cement has been introduced as an esthetic restorative material in pediatric dentistry due to increase awareness for esthetics. These RMGICs were developed to improve the handling and working characteristics of the original glass ionomer formulation. Chemical form of polyacrylic acid component and hybrid layer formed by hydrophilic hydroxyethyl methacrylate (HEMA) of RMGIC causes improved adhesion to the dentin.^16^ Recently, a new composite has been developed by retaining same small particle size of traditional hybrid composites along with a reduced filler content of 20 to 25 wt% resulting in lower viscosity and an lesser elastic modulus than hybrid composites.^17^ In the oral environment, both dissolution of elements and erosion of the nonsoluble components of the restorative material occur. Numerous factors like low pH, acidic foods, ionic composition, ionic strength of saliva and enzymatic attack are important parameters, which may influence the quality and the quantity of the substances released from a restorative material as well as its physical and mechanical characteristics.^18^ The erosive potential of soft drinks has been reported in both *in vivo* and *in vitro* studies. Many erosion studies have employed extremely long immersion regimes ranging from 15 minutes to 72 hours^19,20^ which might not be representative of the normal consumption pattern. There are a number of possible limitations in the above studies as these do not accurately depict the actual impact of the frequency, prolonged and continuous exposure to fruit beverages. The immersion regime followed in the study was similar to the consumption of beverages by an individual.^10^ The pellets were rinsed in normal saline before and after each immersion to neutralize the pH of the beverages.^10^

There was no significant difference in surface roughness in the control group (water) because of the neutral pH of water,^21^ whereas the surface showed more amount of roughness in experimental groups (groups I and II) in both restorative materials.

The number of immersion regimes had a great impact on the surface roughness of resin composite. The excessive surface roughness in resin composite may be due to the following:

- Resin matrix gets softened by some acidic food substances.
- Increased filler content in lower water absorption leading to increased surface degradation.^22^

As the pellets were subjected to more number of immersion time, surface roughness of RMGIC pellets increased because, in acidic solutions, H^+^ ions present in the GIC diffuse into the glassionomer component and it replaces metal cations in the matrix. These cations later diffuse outward and get released to the surface. As the metalions in the matrix decreases more ions surrounding the glass particles will be extracted causing more of dissolution of the GIC. Thus, the outer surface of the GIC becomes more void and rough and protruded undis-solved glass particles. Therefore, prolonged exposure of the glass ionomer of the glass ionomer material to low pH drinks would lead to increased surface roughness.^23^

**Table 4:** **Table 4:** Descriptive statistics on mean surface roughness values of RMGIC following various immersion regime in different media

		*Mild immersion*						*Moderate immersion*						*Severe immersion*					
*Particulars*		*Before*		*After*		*Difference*		*Before*		*After*		*Difference*		*Before*		*After*		*Difference*	
*Group I: Cola drink*																			
Mean		0.423		0.448		0.023		0.425		0.647		0.218		0.432		0.881		0.448	
SD		0.002		0.027		0.026		0.002		0.018		0.017		0.020		0.012		0.021	
Ratio						1.06						1.52						2.07	
p-value*		-		-		p > 0.05 NS		-		-		<0.05 S		-		-		<0.05 S	
*Group II: Fresh fruit juice (citrus)*																			
Mean		0.422		0.424		0.001		0.423		0.552		0.128		0.423		0.701		0.281	
SD		0.001		0.002		0.002		0.002		0.012		0.011		0.001		0.071		0.072	
Ratio						1.0						1.3						1.7	
p-value*		-		-		p > 0.05 NS		-		-		<0.05 S		-		-		<0.05 S	
*Group III: Water (control)*																			
*No immersionregime was followed*																			
Mean		0.422		0.424		0.001													
SD		0.001		0.001		0.001													
Ratio						1.0													
p-value*		-		-		NS													

*p-value: p < 0.001 highly significant (HS); p < 0.05, p < 0.01 significant (S) and p > 0.05 not significant (NS)

**Table 5:** **Table 5:** Intragroup comparison at different concentrations of various media on RMGIC following various immersion regimes

*Groups*		*ANOVA F*		*Mild vs moderate*		*Mild vs severe*		*Moderate vs severe*	
Group I: Cola drink		762.1		p < 0.001 HS		p < 0.001 HS		p < 0.001 HS	
Group II: Fresh fruit juice (citrus)		88.4		p < 0.001 HS		p < 0.001 HS		p < 0.001 HS	

Intragroup comparison: Wilcoxon’s signed rank test; HS: Highly significant

**Table 6:** **Table 6:** Intergroup comparison at different concentrations of various media on RMGIC following various immersion regimes

Differences between groups**		*ANOVA F*		*5.69, p < 0.05 S*		*628.7, p < 0.001 HS*		*217.6, p < 0.001 HS*	
		Groups I-II		p = 0.09 NS		p < 0.001 HS		p < 0.01 S	
		Groups I-III		p = 0.010 NS		p < 0.001 HS		p < 0.001 HS	
		Groups II-III		p = 0.84 NS		p < 0.001 HS		p < 0.001 HS	

**Intergroup comparison: One-way ANOVA, Mann-Whitney test; NS: Not significant; S: Significant; HS: Highly significant

## CONCLUSION

In this study, the various immersion regimes simulated the fruit beverage consumption pattern of an individual, which predisposes the restorations to erosive insult. Thus, it can be concluded that frequent exposure to low pH fruit beverages is directly related to the marginal integrity and surface texture of the materials studied. Such a process may be presumed to occur clinically, which indicates that it is ultimately the patient’s fruit beverage consumption habit that may affect the longevity of the restorations.

## CLINICAL SIGNIFICANCE

The above study highlights the detrimental effects of excessive consumption of fruit juice and soft drinks on existing restorations. Hence, it is incumbent for us as pediatric dentists to counsel our patients following restorative procedures to eliminate the abusive habits associated with consumption of these beverages.
